# Community-Based Control of the Brown Dog Tick in a Region with High Rates of Rocky Mountain Spotted Fever, 2012–2013

**DOI:** 10.1371/journal.pone.0112368

**Published:** 2014-12-05

**Authors:** Naomi Drexler, Mark Miller, Justin Gerding, Suzanne Todd, Laura Adams, F. Scott Dahlgren, Nelva Bryant, Erica Weis, Kristen Herrick, Jessica Francies, Kenneth Komatsu, Stephen Piontkowski, Jose Velascosoltero, Timothy Shelhamer, Brian Hamilton, Carmen Eribes, Anita Brock, Patsy Sneezy, Cye Goseyun, Harty Bendle, Regina Hovet, Velda Williams, Robert Massung, Jennifer H. McQuiston

**Affiliations:** 1 Centers for Disease Control and Prevention, National Center for Emerging and Zoonotic Infectious Diseases, Atlanta, Georgia, United States of America; 2 Centers for Disease Control and Prevention, National Center for Environmental Health, Atlanta, Georgia, United States of America; 3 Centers for Disease Control and Prevention, Epidemic Intelligence Service, Atlanta, Georgia, United States of America; 4 Arizona Department of Health Services, Department of Public Health Services, Phoenix, Arizona, United States of America; 5 Inter-Tribal Council of Arizona Inc., Tribal Epidemiology Center, Phoenix, Arizona, United States of America; 6 Indian Health Service, Office of Environmental Health and Engineering, Phoenix Area Unit, Phoenix, Arizona, United States of America; 7 Indian Health Service, Infection Control Nurse, Phoenix Area Unit, Phoenix, Arizona, United States of America; 8 Tribe B, Department of Health and Human Services, Arizona, United States of America; University of Texas Medical Branch, United States of America

## Abstract

Rocky Mountain spotted fever (RMSF) transmitted by the brown dog tick (*Rhipicephalus sanguineus sensu lato*) has emerged as a significant public health risk on American Indian reservations in eastern Arizona. During 2003–2012, more than 250 RMSF cases and 19 deaths were documented among Arizona's American Indian population. The high case fatality rate makes community-level interventions aimed at rapid and sustained reduction of ticks urgent. Beginning in 2012, a two year pilot integrated tick prevention campaign called the RMSF Rodeo was launched in a ∼600-home tribal community with high rates of RMSF. During year one, long-acting tick collars were placed on all dogs in the community, environmental acaricides were applied to yards monthly, and animal care practices such as spay and neuter and proper tethering procedures were encouraged. Tick levels, indicated by visible inspection of dogs, tick traps and homeowner reports were used to monitor tick presence and evaluate the efficacy of interventions throughout the project. By the end of year one, <1% of dogs in the RMSF Rodeo community had visible tick infestations five months after the project was started, compared to 64% of dogs in Non-Rodeo communities, and environmental tick levels were reduced below detectable levels. The second year of the project focused on use of the long-acting collar alone and achieved sustained tick control with fewer than 3% of dogs in the RMSF Rodeo community with visible tick infestations by the end of the second year. Homeowner reports of tick activity in the domestic and peridomestic setting showed similar decreases in tick activity compared to the non-project communities. Expansion of this successful project to other areas with *Rhipicephalus*-transmitted RMSF has the potential to reduce brown dog tick infestations and save human lives.

## Introduction

Rocky Mountain spotted fever (RMSF) is a severe and potentially fatal tickborne disease caused by the bacterium *Rickettsia rickettsii*. This intracellular bacterium can cause widespread vasculitis resulting in organ failure and death if left untreated, even in previously healthy individuals [1]. Thousands of cases of RMSF are reported annually to the Centers for Disease Control and Prevention (CDC) from across the United States, with the majority of cases originating from the South-Atlantic states [2]. From 2009–2012, the average annual incidence of RMSF in the United States was around 0.9 cases per 100,000 persons [3]. In the United States, RMSF is most commonly transmitted by the American Dog tick (*Dermacentor variabilis*), which is widely distributed east of the Rocky Mountains, and on the California coastline. It is also transmitted by *Dermacentor andersoni* (the Rocky Mountain wood tick) in the western United States. Both of these tick vectors prefer wooded areas and acquire the majority of blood meals from wildlife [4].

The dry, hot Arizona weather is inhospitable to the temperature and humidity requirements of *Dermacentor* ticks, so the risk of contracting RMSF within the state was considered to be low. From 1988 to 2003 only eight cases of RMSF were reported in AZ, most acquired outside the state [5]. However, from 2003 until 2012 the Arizona Department of Health Services (ADHS) reported over 250 cases of RMSF and 19 fatalities, almost all among American Indians without a history of travel [5]. On the three most heavily impacted reservations, the average annual incidence for 2009–2012 was ∼136 cases per 100,000 persons, over 150 times the national average.

Following epidemiologic and ecologic investigations, the dramatic increase in autochthonous RMSF cases in Arizona was linked to transmission by the brown dog tick *(Rhipicephalus sanguineus sensu lato*) [6]. Although this tick species is prevalent throughout the world, the Arizona outbreak was the first time this tick was shown to transmit RMSF infection in the United States. Dogs are the primary host for this tick species at all life stages, and provide both the primary food source for the tick and sites for adult ticks to mate [7]. Dogs are susceptible to infection from *R. rickettsii* and may influence bacterial infection rates in local tick populations [8]. Based on high rates of seropositivity in dogs, the burden of tick parasitism of ticks on dogs, the evidence of *R. rickettsii* bacteremia in some sick dogs with heavy tick burdens, and the abundance of free-roaming dogs in this area, it is likely that dogs serve as a major amplifying host for *R. rickettsii* in this area, although wildlife evaluations have not yet occurred [6], [8]–[14].

In regions where RMSF is primarily associated with *Dermacentor* ticks, RMSF prevention largely centers on human behavior changes and personal protective measures, in order to avoid traditional tick habitats and tick bites. Such activities include wearing light colored clothing, using repellents containing DEET when in contact with wooded areas and high brush, and staying to the inside of trails [15]–[22]. However, such practices are not applicable for avoidance of the brown dog tick, which is found primarily in domestic and peridomestic settings. Because avoidance behaviors do not work in a scenario where constant exposure is likely, preventing *Rhipicephalus*-transmitted RMSF requires control of ticks on dogs (the primary food source) and in the peridomestic environment (the primary location for non-feeding ticks).

In order to determine if brown dog tick control could be attained in a heavily infested community, we designed and evaluated an intervention aimed at killing ticks on dogs and in the peridomestic environment using properly timed environmental acaricide application and long-acting tick collars for dogs.

## Methods

### Setting

**Figure 1:**
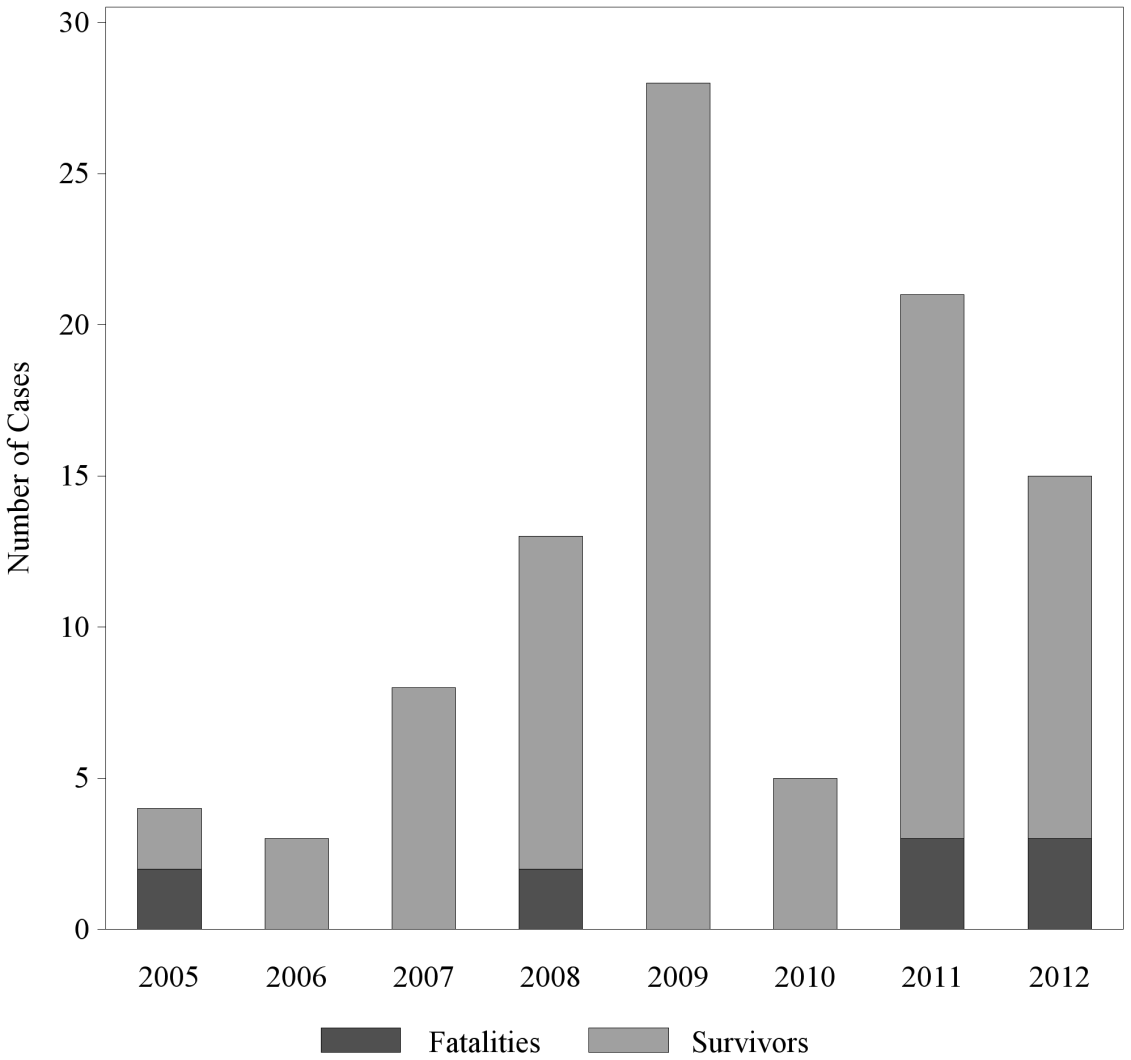
Human cases of Rocky Mountain spotted fever on Reservation B as reported by the Arizona Department of Health Services.

The pilot tick prevention project, called the RMSF Rodeo, was conducted on Reservation B in Arizona, which is home to ∼10,000 individuals. This reservation is principally located in a high altitude desert zone receiving less than 18 inches of precipitation annually [23]. This area produces temperatures that are warm enough (average annual high 76° F, average annual low 47° F) to sustain brown dog tick populations year round [24], [25]. Previous tick control interventions in this community included seasonal provision of granular acaricide for yards and application of Zodiac tick collars containing the active ingredient propoxur (labeled for 5 months activity) to some houses on the reservation, or upon request to local public health authorities; however, these efforts did not provide sufficient control as reports of human cases continued to increase (figure 1). Because most dogs on the reservation were free-roaming, tick control strategies had to be made at a community-level, rather than the household level in order to be effective.

The community within Reservation B that was selected for participation in the RMSF Rodeo had been highly impacted by RMSF, including two fatalities that occurred just prior to the start of the project. The community contained ∼600 (581 in 2012 and 571 in 2013) occupied homes, and was geographically isolated from other neighborhoods by open desert, roads, and a river basin.

### Program intervention

The RMSF Rodeo was piloted to assess the efficacy of properly timed environmental acaricide treatment of home sites, treatment of dogs with long-acting tick collars, and improved access to pet care practices (tethering, spay/neuter). The program was designed to have repeated contact with participating households and dogs during times of peak tick activity in order to ensure that there were no lapses in environmental or veterinary treatments.

RMSF Rodeo activities were conducted April 2012 – September 2013 and consisted of two distinct phases. Phase 1 (April–August 2012) focused on immediate control of ticks, including:

- 1) Registration of homes and dogs. Homes were registered beginning in April 2012. Participating homes were offered tie-out stakes for dogs, and dogs were given a nylon collar with a numbered registration tag.
- 2) Application of long-acting tick collars. Seresto tick collars, containing 4.5% flumethrin and 10% imidacloprid, (estimated to control ticks for 7 to 9 months) [26] were placed on all participating dogs at the time of registration. Seresto collars were donated by Bayer during 2012 and used only with dog owner permission.
- 3) Application of liquid acaricide. Bayer Advanced Multi-insect Killer, containing 0.4% Beta-cyfluthrin and 0.7% imidaclorprid was applied by trained volunteers to the yards of all participating homes, and was re-applied at one month intervals May–August 2012. The entire perimeter of each home was treated, from 5 feet out from the side of the home, 3 feet up the wall of the home, and anywhere on the premises that dogs were reported to frequent (ex. dog houses, under porches or houses). Pest management professionals treated homes where indoor tick presence was reported by homeowners.
- 4) Regular follow-up. RMSF Rodeo homes were visited once a month for pesticide application, collar replacement, when necessary, and routine monitoring of tick populations on dogs.
- 5) Dog population control. Participating households were provided with information about free spay-neuter opportunities offered by the program during June 2012 and were encouraged to have their dogs spayed or neutered.

Phase 2 of the RMSF Rodeo (March–September 2013) focused on sustained control of ticks, consisting of:

- 1) Reapplication of long-acting tick collars. Seresto tick collars were replaced on all participating dogs in March 2013.
- 2) Referral of home sites for environmental acaricide treatment. Participating homes with observed tick activity documented during bimonthly checks were referred for acaricide treatments using the same product and application methodology as Phase 1.
- 3) Regular follow-up. RMSF Rodeo homes were visited once every two months for collar replacement, when necessary, and routine monitoring of tick populations on dogs.
- 4) Dog population control. Free monthly mobile spay and neuter clinics provided by the tribe using one-time grant funds and were advertised during RMSF Rodeo activities.

Between Phase 1 and Phase 2 of the RMSF Rodeo (September 2012 – March 2013), there were no formal Rodeo project activities, although the tribe continued to distribute Zodiac tick collars and treat homes upon request. Throughout the two years, the tribal animal control program increased capacity to collect and remove stray dogs across the reservation. Education about tick control and RMSF prevention were emphasized to all communities, although the RMSF Rodeo allowed for more frequent interaction with homeowners. Team members responsible for acaricide application, dog collaring and tick assessments were trained prior to each interaction to limit the amount of inter-operator variability. Teams typically consisted of 15 members (range 12–25), with 2–3 people at each location, roughly equating to ∼10,000 person-hours in the field in 2012 and 2013 (not including day-to-day animal control practices). Data associated with the routine monitoring of the dogs in both phases of the project were recorded on paper registers and were entered into Microsoft Excel databases following each visit.

### Tick activity outcomes

Five homes within the RMSF Rodeo community were followed throughout the project to monitor tick burden in the environment. Carbon dioxide (CO_2_) tick traps, consisting of a 36 inch by 36 inch square of white flannel were placed in three locations at each of the five homes. These traps were set once a month from May through August in Phase 1, and in March and May of Phase 2, but were discontinued thereafter. Tick traps were collected after 4 hours. The trapped ticks were killed by freezing, sent to the medical entomology laboratory, where they were characterized as *R. sanguineus sensu lato* and counted by life stage. Ticks observed on dogs were not characterized at each observation. Since *R. sanguineus sensu lato* ticks were the only tick species observed in this and other environmental evaluations in the area, all morphologically consistent ticks were assumed to be of the same species and lineage [6], [11].

Dogs registered in the RMSF Rodeo were tagged at registration to validate their participation and provide a means of identification. Dogs that could be caught and examined (both restrained and free-roaming dogs) were checked on a monthly basis in Phase 1 and bimonthly in Phase 2 for maintenance of their tick collar and visual tick inspections throughout the program. Dogs were examined for ticks on the ears, face, and between the toes. Visual tick inspections were categorized as: A. zero ticks visible, B. 1–20 ticks visible and C.>20 ticks visible.

### End-of-Phase evaluations

At the end of each phase of the project (August 2012 and September 2013), systematic evaluations were performed to compare tick levels in both RMSF Rodeo and Non-Rodeo communities on Reservation B, and to compare practices surrounding RMSF prevention and tick control. During 2013, a reservation-wide tick prevention program was implemented in the Non-Rodeo areas, so End-of-Phase 2 evaluation results are more limited in scope and evaluations of RMSF Rodeo success are best compared with the Non-Rodeo areas in 2012 (when no intervention was applied).

To assess the effectiveness of the project and identify possible interventions according to respondents, the Rodeo community and three Non-Rodeo communities were surveyed about knowledge, attitudes, and practices surrounding RMSF, dog ownership, and animal control. Households were stratified by neighborhood, and a proportionate stratified sample of households was drawn without replacement. Households in Non-Rodeo communities were over-sampled in anticipation of lower participation. Teams travelled house-to-house administering a questionnaire to any adult member of the household who consented to participate. Electronic questionnaires were designed and data were compiled using MR Interviewer software version 5.6 [27]. Participant responses were recorded on secured tablets or paper surveys, as availability permitted. Electronically entered data were encrypted and synchronized nightly with secure servers maintained by the CDC.

### Statistical analysis

Data were analyzed using SAS version 9.3 [28]. Univariate analyses were performed including 95% confidence intervals, and chi-squared tests were used to evaluate the differences in categorical variables. Significance was considered at alpha <0.05. End-of-Phase evaluation data were weighted to account for sampling methodologies and differential survey response by neighborhood. Weighted frequencies and 95% confidence intervals are reported for End-of-Phase evaluation data. In a sub-analysis of 2013 data from homes with dogs, risk ratios were estimated using PROC GENMOD log-binomial model to assess associations between key risk factors and tick burden.

### Human case data

Human cases of RMSF meeting a confirmed or probable case definition are reported to the Arizona Department of Health Services (ADHS) annually [29]. Confirmed cases provide the strongest evidence a case is RMSF, and include cases diagnosed by polymerase chain reaction (PCR) assay and antibody titers with a fourfold change between paired sera. Probable cases are based on single antibody-positive tests or paired tests showing less than a fourfold change. In April 2014, RMSF cases reported from Reservation B with onset between April 2010 and April 2014 were retrospectively categorized into RMSF Rodeo and Non-Rodeo communities by local public health authorities based on last known location of residence. Incidence was then calculated using average case counts for the 2 years before (April 1, 2010 – March 31, 2012) and the 2 years after (April 1, 2012 – March 31, 2014) the start of the RMSF Rodeo. Population size was estimated based on reported number of members in household among RMSF Rodeo participants using End-of-Phase 1 survey data. Population size for Non-Rodeo communities was then calculated using the remaining difference between RMSF Rodeo population and 2010 census estimates [30].

### Ethics statement

Approval for this prevention project was obtained from the Reservation B tribal council by CDC prior to the start of activities in 2012. End-of-Phase surveys were reviewed by the CDC Human Subjects Protection Office and were deemed exempt from CDC Institutional Review Board on a non-research basis. All individuals interviewed were at least 18 years of age and the survey posed minimal risk to participants. Written informed consent was obtained from all participants and no personally identifiable information was tied to survey responses.

## Results

Within the RMSF Rodeo community, 98% (576/582) of occupied households in 2012, and 99% (558/571) of occupied households in 2013 participated in the intervention project. Although defining a precise number of dogs was difficult due to births, deaths, loss, and transfer of ownership; we estimate that roughly 1000 dogs were managed within the RMSF Rodeo community each year, but that number fluctuated by month.

### Observed ticks

**Figure 2:**
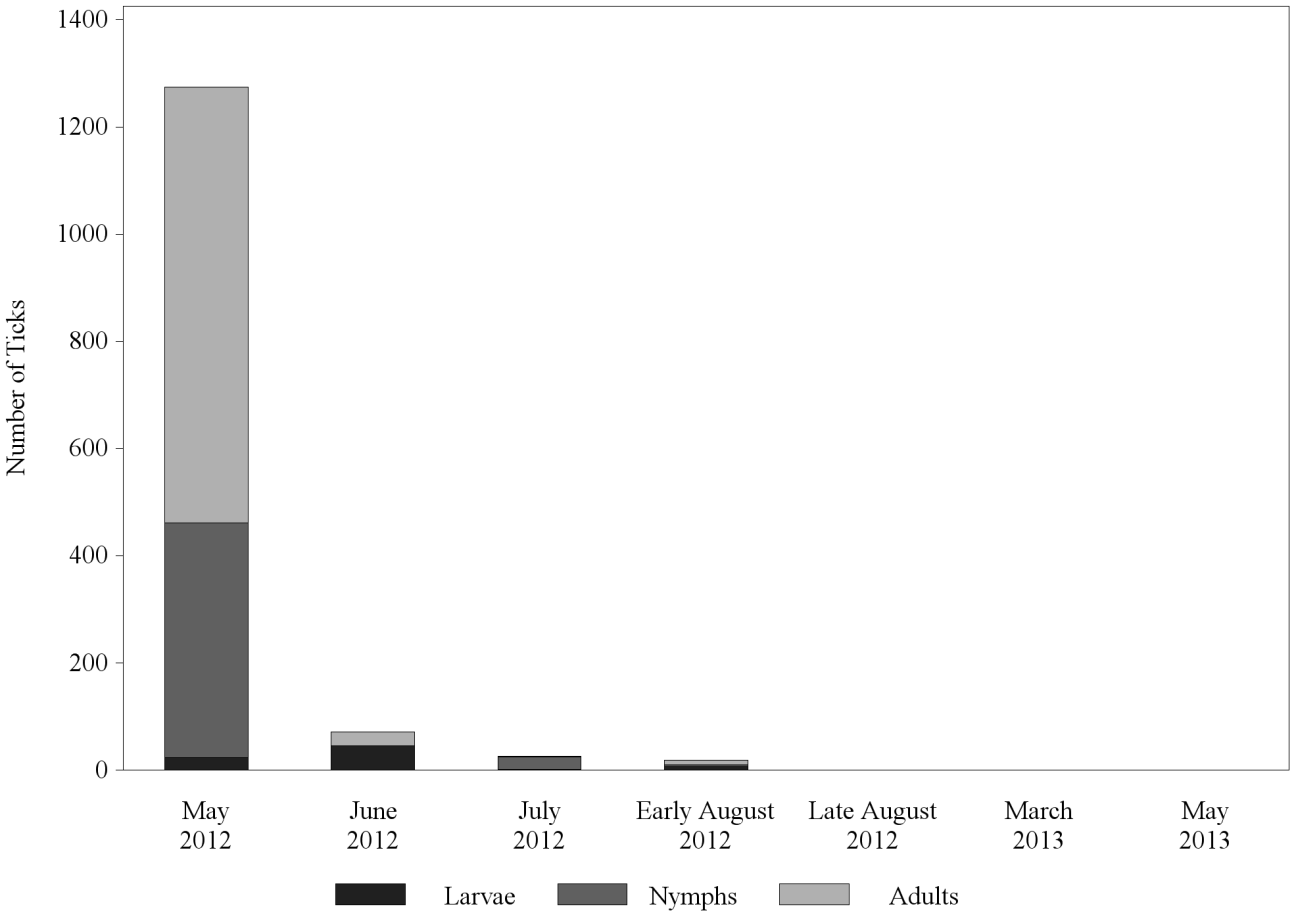
Observed ticks by life stage in CO_2_ traps in the RMSF Rodeo community, n = 5 homes, 3 traps per home.

Substantial numbers of ticks were captured in the CO_2_ tick traps in May 2012 (n = 1274). Tick numbers decreased drastically in June (n = 54), with continuing decreases in the months to follow; no ticks were observed in environmental traps by the end of Phase 1 (figure 2). Tick traps were set again in March and May of 2013, but no ticks were captured at any locations. Tick trapping was discontinued as it was no longer sensitive to measuring tick burden in the environment.

**Figure 3:**
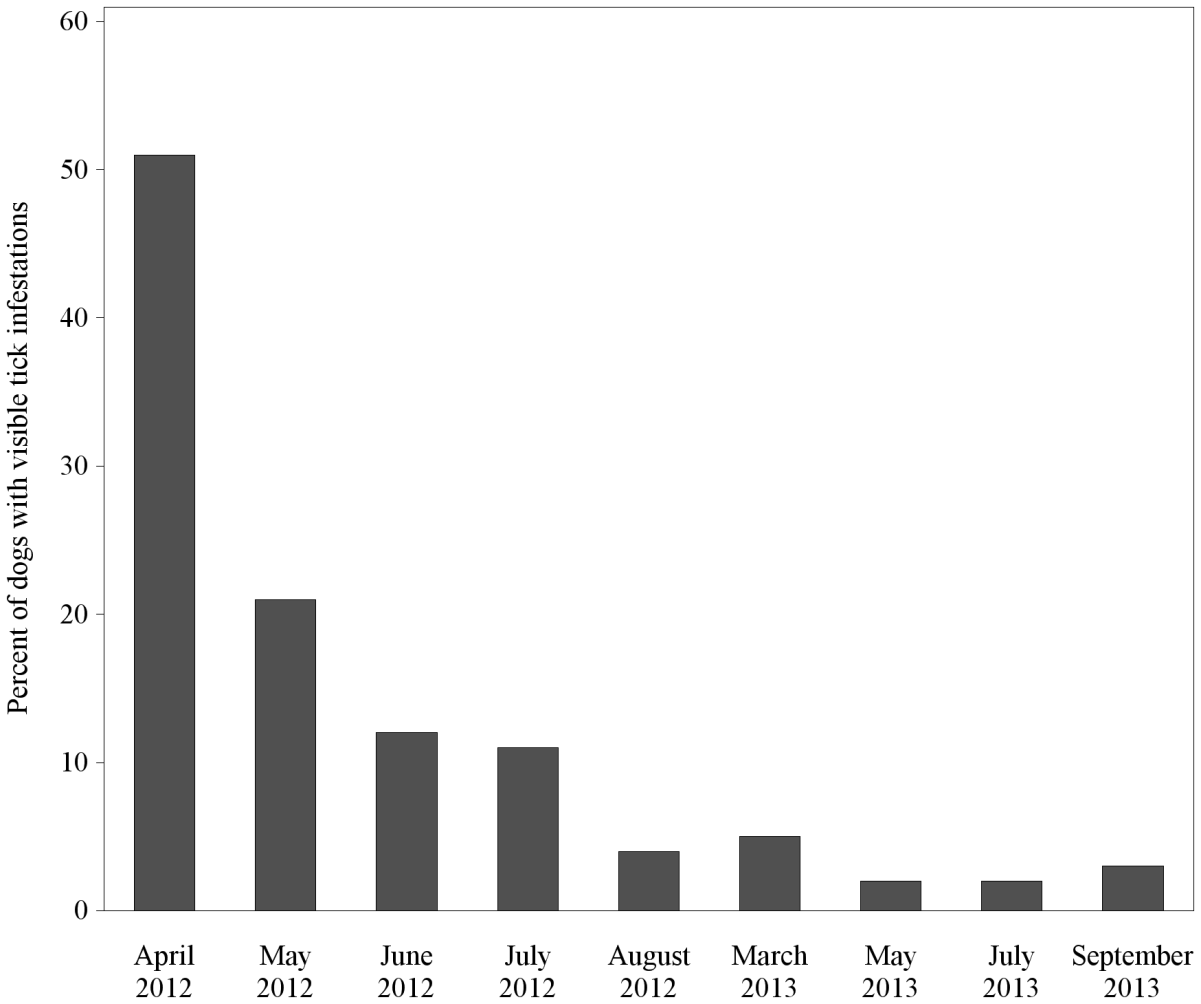
Percent of dogs registered in the RMSF Rodeo with visible tick infestations, assessed during routine monitoring.

Dogs belonging to households participating in the RMSF Rodeo were monitored over time for visible tick infestations. Each data point represents a cross-sectional assessment of observed ticks on dogs in the project area, as not all dogs were seen during every visit. Fifty-one percent of registered dogs had visible tick infestations at the start of the RMSF Rodeo in April 2012; this decreased to <4% of dogs with ticks visible in August 2012 (figure 3). During Phase 2 of the project, <6% of dogs in the RMSF Rodeo community had ticks visible in March, and this level was sustained through the end of the project in September 2013.

### End-of-Phase evaluations

End-of-Phase evaluations allowed for the comparison of RMSF Rodeo and Non-Rodeo communities using data from households participating in the survey. Selected characteristics of these populations can be seen in table 1; no substantial differences between the demographics of these households were detected. The evaluation did show differences between the Rodeo and Non-Rodeo communities in terms of reported dog care practices. Restraint of dogs was more common in RMSF Rodeo area than Non-Rodeo areas in 2012; with households in the Rodeo area being 30% more likely to restrain their dogs than households in the Non-Rodeo area. Although there was a slight increase in number of people reporting never restraining their dog(s) in the RMSF Rodeo area between 2012 and 2013, the change was not statistically significant (p = 0.4454). Spay and neuter practices were more common in the RMSF Rodeo area than in the Non-Rodeo areas in 2012 (RR = 1.7 neutered, RR = 2.7 spayed), which is not surprising as these services were offered more aggressively in this area in 2012. However, the use of spay and neuter services seems to have continued in 2013 with 32% of RMSF Rodeo dog owners reporting they had at least one dog “fixed” in the last year. Increased utilization of spay and neuter services will provide long-term reduction in stray and unwanted dog populations in the area, leading to fewer blood meals for the brown dog tick.

**Table 1:** Respondent characteristics from selected populations in the program evaluation survey.

	Non-Rodeo 2012	Rodeo 2012	Rodeo 2013
**Female**	45.2% (39, 51)	62.6% (56, 69)	51.9% (45, 59)
**Age**			
**18–25 yrs**	19.2% (14, 24)	15.2% (10, 20)	14.8% (10, 20)
**26–50 yrs**	43.1% (37, 49)	54.2% (47, 61)	51.3% (45, 58)
**51+ yrs**	37.8% (32, 44)	29.5% (23, 36)	33.9% (28, 40)
**Number of dogs per household**	mean = 1.6 (range 0–13)	mean = 1.8 (range 0–10)	mean = 1.8 (range 0–13)
**Number of kids per household**	mean = 2.0 (range 0–8)	mean = 2.2 (range 0–10)	mean = 2.2 (range 0–9)
**Response rate of sampled homes**	234/315 (74%)	192/280 (69%)	199/280 (71%)
**Restraint practices among dog owners**			
**Always**	28.3% (21, 35)	38.9% (30, 48)	30.0% (23, 37)
**Sometimes**	26.7% (20, 34)	34.9% (27, 43)	41.5% (34, 49)
**Never**	45.0% (37, 53)	26.2% (19, 34)	28.5% (21, 36)
**Number of dogs owned**			
**Greater than 2 dogs**	24.8% (21,33)	25.5% (18, 33)	23.1% (16, 31)
**1 or 2 dogs**	36.8% (29, 45)	39.6% (32, 47)	49.3% (43, 56)
**zero dogs**	38.5% (33,44)	34.9% (29, 40)	27.6% (22, 33)
**At least 1 male dog fixed**	11.4% (6,18)	30.5% (26, 39)	NA
**At least 1 female dog fixed**	24.7% (17, 36)	41% (33, 52)	NA
**At least 1 dog fixed in the last year**	NA	NA	31.9% (25, 39)
**Check kids for ticks among parents**	79.4% (74, 85)	79.5% (73, 86)	84.4% (79, 90)

Reported as weighted percent frequency (95% confidence interval), unless otherwise indicated.

Visible tick counts on dogs were also observed among surveyed households with dogs, and can be compared between RMSF Rodeo and Non-Rodeo communities (table 2). Over 99% of dogs in the RMSF Rodeo communities were tick-free in 2012, compared to only 36% of dogs in the Non-Rodeo communities. In 2013, 98% of dogs had no visible tick infestation in the RMSF Rodeo area. This finding is not statistically different from the 2012 End-of-Phase 1 numbers in the RMSF Rodeo area (Fisher's exact p = 0.1842), suggesting control was sustained during Phase 2.

**Table 2:** Observed tick counts on dogs during the End-of-Phase evaluations.

	2012		2013
	Non-Rodeo	Rodeo	Rodeo
**Zero ticks**	36.1% (28, 44)	99.2% (98, 100)	97.7% (95, 100)
**1**–**20 ticks**	32.2% (24, 40)	0.8% (0, 2)	2.4% (0, 5)
>**20 ticks**	31.7% (24, 40)	0%	0%

Reported as weighted percent frequency (95% confidence interval).

**Figure 4:**
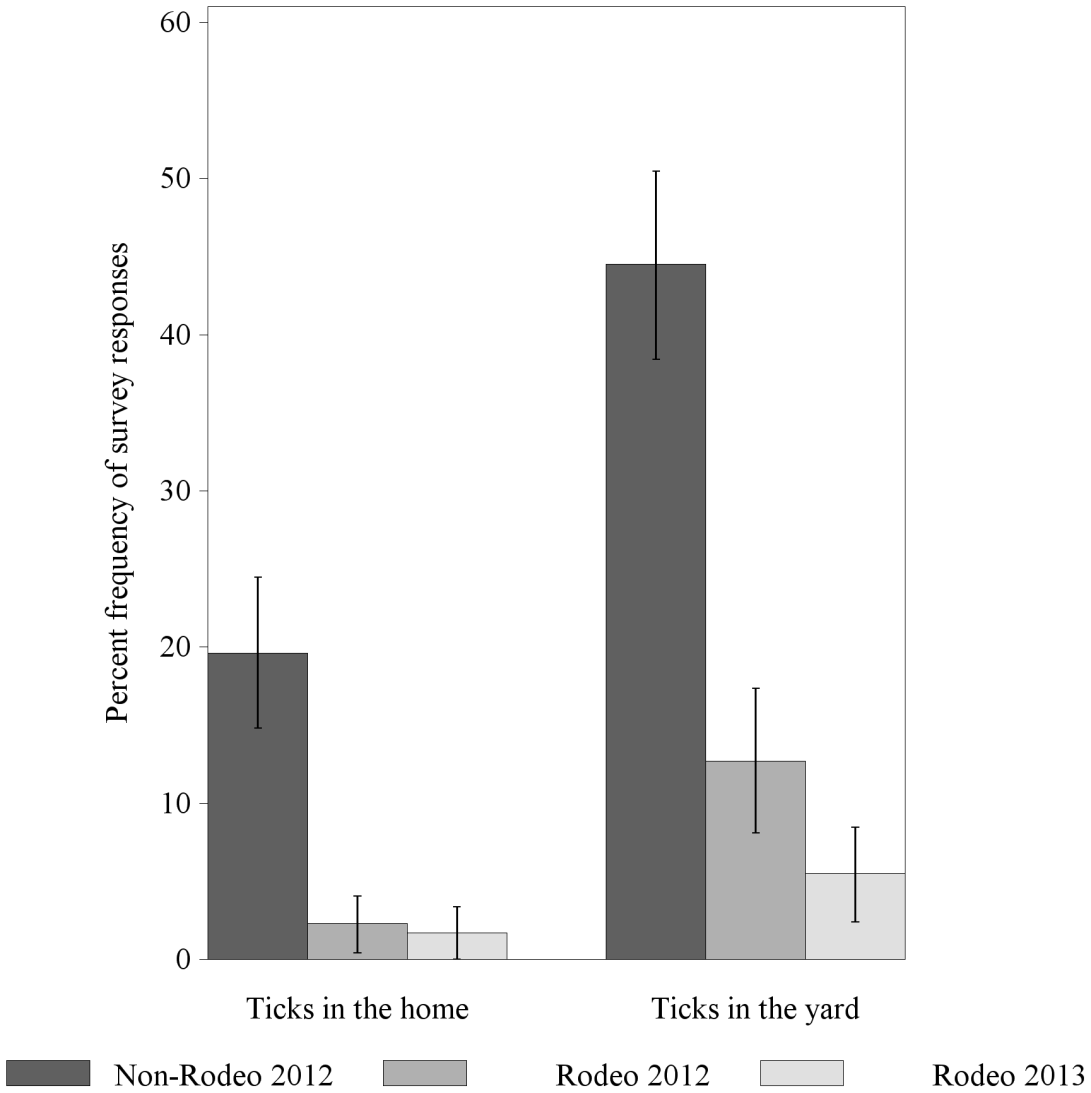
Homeowner reports of tick activity, assessed during the End-of-Phase evaluations.

The End-of-Phase evaluations also collected homeowner reports of ticks inside their house (domestic) or in their yard (peridomestic) (figure 4). In 2012, 20% of people reported having seen ticks in their homes in the last month in the Non-Rodeo area, compared with only 2% in the RMSF Rodeo community. Similar disparate reports were seen in yard infestations: 45% of people in the Non-Rodeo area reported seeing ticks, compared with 13% in the RMSF Rodeo area. Decreases in reported tick activity were observed during the End-of-Phase 2 evaluation: only 2% of RMSF Rodeo households reported seeing ticks in the home, and only 6% reported seeing ticks in yards.

In order to address factors associated with observed tick activity, a sub-analysis was performed of households in 2013 owning at least one dog (table 3). Statistically significant factors associated with the presence of tick infestations included lack of a Seresto collar (RR = 5.4, p<0.05), and having more than 2 dogs (RR = 1.6, p<0.05). Always restraining your dog was protective (RR = 0.55, p<0.05), and never restraining was associated with a slight increase (RR = 1.2, p = 0.09) in tick infestation compared to sometimes restraining, although the latter was not statistically significant. Risk factors were evaluated for confounding using logistic regression and no significant confounding was observed.

**Table 3:** Weighted frequencies and risk ratios associated with tick activity observed on dogs as part of the End-of-Phase evaluation in 2013.*

	Dogs with ticks	Dogs without ticks	Risk ratio (95% CI)
**Seresto collar**			
**No Seresto collar observed**	85.5% (73, 98)	42.5% (34, 51)	5.4 (4.0, 7.5)
**Seresto collar observed**	15.5% (2, 27)	57.5% (49, 66)	ref
**Number of dogs**			
**Greater than 2 dogs**	46.3% (28, 65)	32.1% (25, 40)	1.6 (1.3, 2.0)
**Fewer than 2 dogs**	53.7% (35, 72)	67.9% (60, 75)	ref
**Restraint practices**			
**Always restrain**	19.2% (5, 34)	36.1% (28, 44)	0.55 (0.40, 0.74)
**Never restrain**	41.8% (23, 60)	28.8% (21, 36)	1.2 (0.97, 1.5)
**Sometimes restrain**	38.9% (21, 57)	35.2% (28, 43)	ref

*This analysis only relates to homes with at least one dog.

**Figure 5:**
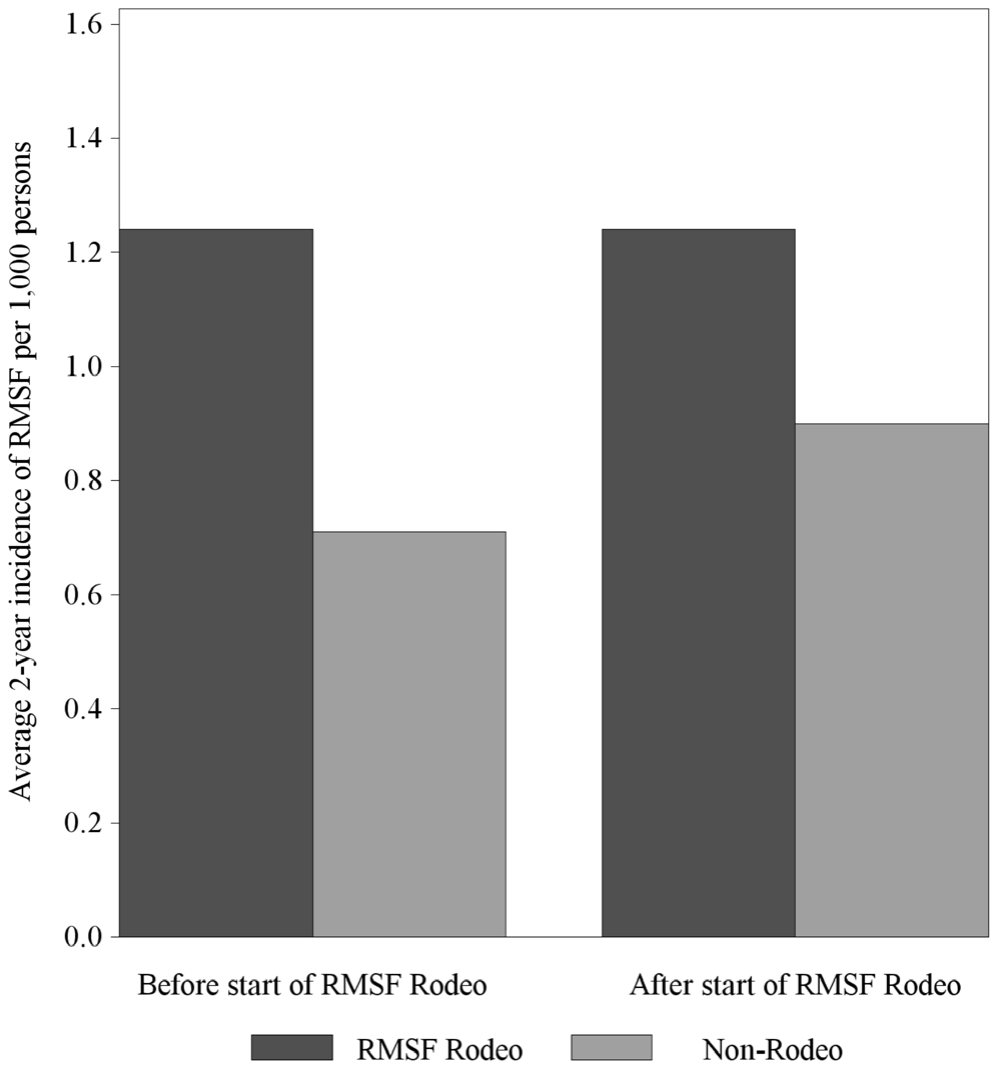
Human case incidence of Rocky Mountain spotted fever in the RMSF Rodeo community and Non-Rodeo communities before and after the start of the RMSF Rodeo in April 2012.

Of reported human cases of RMSF on Reservation B, 62% of the cases in this four-year span met a probable case definition and 38% were considered confirmed. Average annual incidence of human cases of RMSF was estimated to be 1.2 cases per 1000 persons in both the RMSF Rodeo community and in the Non-Rodeo communities prior to the start of the RMSF Rodeo in April of 2012. In the two years following, average incidence in the RMSF Rodeo community decreased by 43% to 0.71 cases per 1000 persons. Cases also decreased in the Non-Rodeo communities, to 0.90 cases per 1000 persons, a decrease of 27% (see figure 5).

## Discussion

The RMSF Rodeo tick prevention project successfully decreased tick levels within this tribal community, and maintained low levels of ticks for a period of at least two years. In the first year, environmental tick control methods combined with long-acting tick collars on dogs produced substantially fewer domestic and peridomestic infestations in the RMSF Rodeo community compared to the Non-Rodeo communities, as evidenced by observed tick activity on dogs, environmental CO_2_ traps, and homeowner reports of sighted tick activity. Once tick control had been achieved during Phase 1 of the RMSF Rodeo, using the combined environmental and veterinary tick control, tick populations were sustained at very low levels during Phase 2 of the RMSF Rodeo using tick long-acting collars alone.

We believe that the success of the RMSF Rodeo was due to the tailoring of interventions around this tick vector and its particular habits. The start of the project in March-April of each year was timed to correspond with human RMSF surveillance data showing increased cases during those months, presumably due to increased tick activity [7], [31], [32]. Use of properly timed environmental acaricides with repeated applications during Phase 1 allowed for the control of hatched ticks in the environment at several points during the year. The simultaneous placement of a long-acting tick collar prevented community dogs from becoming both the food and reproductive resource for this tick vector. Evidence indicates that at the individual dog level presence of a tick collar was highly associated with absence of ticks, and in 2013, un-collared dogs were more than five times as likely to have visible tick infestation as collared dogs. This suggests that presence of the collar is a pivotal factor in the individual and likely community-wide control of ticks. Always restraining dogs was seemingly protective of observed tick activity, and never restrained dogs were at increased risk of having observable ticks. Previous studies have shown that restrained dogs are more likely to have tick infestations than unrestrained dogs, presumably because of high levels of local environmental infestation, and because they are easy prey for meal-seeking ticks [33]. However in this case, where concurrent yard treatments were used in addition to collar treatments, we believe unrestrained dogs were more likely to travel to untreated (non-peridomestic) areas or interact with other, non-treated dogs, thereby increasing their risk of tick infestation.

Another important factor in the success of the project was the strong degree of support for the RMSF Rodeo within the community, as demonstrated by the high rate of household enrollment. Households were visited multiple times and registration was repeatedly encouraged in order to procure this high rate of participation, which was crucial in the project's ultimate success.

The finding that ticks were controlled across the community during Phase 2 of the project using tick collars alone is very promising. Even though Phase 2 of the project included a contingency for acaricide treatment in the project area for households where tick activity was observed, this was necessary in <5% of households in the RMSF Rodeo community, suggesting that the tick collar alone was sufficient in preventing the majority of visible tick infestations on dogs once environmental burden was reduced. This method enables more targeted and cost-efficient strategies of tick control, interceding on the primary host, and will reduce the amount of pesticides necessary in the environment.

The RMSF Rodeo project did not evaluate the efficacy of a tick collar alone during Phase 1 of the project, and we do not recommend this option for communities with high environmental tick loads. We believe rapid and immediate killing of ticks in the environment is essential to reduce RMSF risks in highly impacted communities. There is also a possible risk: if collars are used without controlling ticks in the peridomestic environment, meal-seeking ticks may be inclined to parasitize other unprotected animals in the immediate area including humans. Thus, during a period of initial tick control, we recommend a combined approach that includes environmental treatments.

The RMSF Rodeo was not designed to compare the efficacy of different tick control products. Seresto collars were selected because they provided a visible marker of dog treatment, were easy to apply, and represented the longest-acting product of this type with market approval. Similarly, the Bayer Advanced environmental acaricide was used because it was donated by the company, but also because it could be purchased and applied by homeowners without special licenses in the future. It is possible that similar tick control could also be achieved using different products; however, product longevity and effectiveness should be considered when selecting products.

This pilot project is subject to some limitations. Survey answers are subject to recall bias and interpretation, as well as perceived pressures to provide socially acceptable answers. Respondents in the RMSF Rodeo community may have felt a greater need to provide responses which inflate the project's success out of courtesy to the interviewer, which would bias our results away from the null. Neighborhoods were separated into intervention (RMSF Rodeo) and non-intervention (Non-Rodeo) communities; however, in some unusual cases we found RMSF Rodeo collars on dogs in Non-Rodeo communities, as a result of sharing of products between family members or translocation of dogs. While RMSF Rodeo and Non-Rodeo communities were geographically isolated from one another, unrestrained dogs may also have traveled outside of their intervention area or been translocated by human activity. These occurrences could result in spill-over between intervention and non-intervention communities, and may have introduced a bias towards the null hypothesis. Despite best efforts, dogs had the potential to be lost or duplicated among program records. Longitudinal analysis of all enrolled dogs was not possible as dogs were continually enrolled, died, or changed locations and identification tags were lost and duplicated; therefore, only cross-sectional data are reported. Our final limitation was the inability to track the Non-Rodeo areas for a second year to serve as a control group. Due to the overwhelming success of the RMSF Rodeo in 2012, the tribe implemented a modified tick control program in Non-Rodeo areas in 2013.

Reducing RMSF cases among tribal residents is the primary goal of the RMSF Rodeo. Control of tick activity in domestic and peridomestic locations is expected to reduce the risk of human exposure to *R. rickettsii* carrying ticks, but measuring the effects on human incidence is difficult to document. Before the start of the RMSF Rodeo there was high incidence of RMSF among residents of the RMSF Rodeo and Non-Rodeo communities. In the two years following the start of the intervention, decreases in human incidence were observed across the reservation; however a more substantial decrease was noted in the RMSF Rodeo community. The significance and attribution of the observed decrease, however, is uncertain, and we believe that it will take several more years to fully measure the impact of this project. Reported human cases of Rocky Mountain spotted fever fluctuate widely year to year, and are influenced by human exposure patterns and testing and reporting practices [2]. Due to the relatively new emergence of this disease in Arizona, trends in disease occurrence are not yet well established, and may be influenced by factors such as rainfall and temperature, which vary from year to year [34]. Tribal residents frequently travel in between communities and may be exposed to ticks elsewhere on the reservation, so location of residence does not necessarily correspond to location of exposure. Reduction in human cases in the Non-Rodeo areas may be due to annual variation, but could also be ascribed to reservation-wide RMSF control efforts including improved animal control, provision of spay and neuter services and reservation-wide collaring and environmental acaricide treatment beginning in 2013. Finally, diagnosis of RMSF was frequently based on detection of antibodies in single serum samples, and in high-incidence areas a high percentage of the population may be antibody-positive from past exposures, rather than new acute infections [35]. We plan to continue tracking human cases using surveillance data and working with physicians and local public health authorities to improve the case confirmation process to better document human cases of RMSF in these communities and evaluate long-term changes in incidence.

Since the development of this project, the need for *Rhipicephalus*-transmitted RMSF prevention has only grown. There are now six American Indian reservations in Arizona with documented human cases of RMSF, placing more than 350,000 American Indians at risk for this deadly disease [30]. The evidence obtained in this project has been used to generate RMSF prevention strategies for other Arizona tribes considering RMSF prevention programs, and has informed the substantial effort and vigilance that must go along with continued RMSF prevention within the state.

While the 2-year RMSF Rodeo program achieved a remarkable degree of tick control, it is worth noting that ticks were not completely eliminated in the RMSF Rodeo community. Tick control efforts will need to be maintained in coming years in order to keep the risk of tick bite and RMSF reduced in this community. It is unlikely that a full elimination scheme can be achieved for such a ubiquitous pest. However, it is the hope that adequate tick control in the environment and on animals will decrease the opportunities for human illness, and, when coupled with supportive care from well-trained physicians, cases can be caught sooner and deaths prevented.
